# Variation in *optineurin* (*OPTN*) allele frequencies between and within populations

**Published:** 2007-02-02

**Authors:** Rosa M. Ayala-Lugo, Hemant Pawar, David M. Reed, Paul R. Lichter, Sayoko E. Moroi, Michael Page, James Eadie, Veronica Azocar, Eugenio Maul, Christine Ntim-Amponsah, William Bromley, Ebenezer Obeng-Nyarkoh, A. Tim Johnson, Theresa Guckian Kijek, Catherine A. Downs, Jenae M. Johnson, Rodolfo A. Perez-Grossmann, Maria-Luisa Guevara-Fujita, Ricardo Fujita, Margaret R. Wallace, Julia E. Richards

**Affiliations:** 1 Department of Ophthalmology and Visual Sciences, The University of Michigan, Ann Arbor, MI; 2 Universidad Nacional de Asunción, Hospital San Pablo, Asunción, Paraguay; 3 Universidad Catolica de Chile, Santiago, Chile; 4 University of Ghana, Accra, Ghana; 5 Center for Human Genetics, Bar Harbor, ME; 6 Department of Ophthalmology and Visual Sciences, the University of Iowa, Iowa City, IA; 7 Centro de Genética y Biología Molecular, Facultad de Medicina, Universidad de San Martín de Porres, Lima, Perú; 8 University of Florida, Gainesville, FL; 9 Department of Epidemiology, the University of Michigan, Ann Arbor, MI

## Abstract

**Purpose:**

To evaluate the extent to which mutations in the *optineurin* (*OPTN*) glaucoma gene play a role in glaucoma in different populations.

**Methods:**

Case-controlled study of *OPTN* sequence variants in individuals with or without glaucoma in populations of different ancestral origins and evaluate previous *OPTN* reports. We analyzed 314 subjects with African, Asian, Caucasian and Hispanic ancestries included 229 cases of primary open-angle glaucoma, 51 cases of juvenile-onset open-angle glaucoma, 33 cases of normal tension glaucoma, and 371 controls. Polymerase chain reaction-amplified *OPTN* coding exons were resequenced and case frequencies were compared to frequencies in controls matched for ancestry.

**Results:**

The E50K sequence variant was identified in one individual from Chile with normal tension glaucoma, and the 691_692insAG variant was found in one Ashkenazi Jewish individual from Russia. The R545Q variant was found in two Asian individuals with primary open-angle glaucoma; one of Filipino ancestry and one of Korean ancestry. In addition to presenting *OPTN* allele frequencies for Caucasian and Asian populations that have been the subject of previous reports, we also present information for populations of Hispanic and black African ancestries.

**Conclusions:**

Our study contributes additional evidence to support the previously reported association of the *OPTN* E50K mutation with glaucoma. After finding an additional 691_692insAG *OPTN* variant, we can still only conclude that this variant is rare. Combined analysis of our data with data from more than a dozen other studies indicates no association of R545Q with glaucoma in most populations. Those same studies disagree in their conclusions regarding the role of M98K in glaucoma. Our analysis of the combined data provides statistically significant evidence of association of M98K with normal tension glaucoma in Asian populations, but not in Caucasian populations; however, the validity of this conclusion is questionable because of large differences in allele frequencies between and within populations. It is currently not possible to tell how much of the underlying cause of the allele frequency difference is attributable to demographic, technical, or ascertainment differences among the studies.

## Introduction

The optic neuropathy called glaucoma is the second most common cause of bilateral blindness in the world [1]. The most frequent form of glaucoma is open-angle glaucoma (OAG), which can occur as adult-onset primary open-angle glaucoma (POAG) or juvenile-onset primary open-angle glaucoma (JOAG). Prevalence of OAG has been measured at higher frequencies in individuals of African ancestry than in those of European or Asian ancestry [2]. Although the most common form of OAG in the US involves elevated intraocular pressure (IOP), OAG also develops in individuals whose IOP is never observed outside of the normal range (normal tension glaucoma, NTG). Associated risk factors for OAG include race (i.e., population genetic factors, particularly ethnic ancestry), a family history of glaucoma, increasing age, and an IOP elevated above the normal range.

During the last decade, genetic mapping and cloning experiments have demonstrated that glaucoma has substantial genetic components [3]. Among the more than one dozen mapped glaucoma loci, 11 GLC1 loci cause OAG [3], two GLC3 loci cause congenital glaucoma [4,5], and the remaining known loci are responsible for secondary and developmental forms of glaucoma [3]. Additional genetic risk factors for differential severity of OAG have been reported [3].

Mutations in the myocilin (*MYOC*) gene at the GLC1A locus are found in 2.6-4.3% of POAG cases [6] and up to one-third of the familial JOAG cases [7]. There has not yet been a follow-up on a recent report that mutations in WD repeat domain 36 (WDR36) that occur in cases of POAG that map to the GLC1G locus on chromosome 5 [8].

Rezaie et al. [9] described mutations in the *optineurin* (*OPTN*) gene, located within the GLC1E interval at 10p15-p14 [10], in 16.7% of families from a predominantly NTG population. The original report of *OPTN* involvement in glaucoma presented three likely disease-causing variants designated E50K, 691_692insAG, and R545Q, and one proposed risk factor M98K [9]. Further studies find association of some *OPTN* alleles with OAG, but others report no evidence of association of OAG with those same alleles [11-24].

In this paper, we present new data on our *OPTN* mutation screening of 314 open-angle glaucoma patients who have either POAG, JOAG, or NTG, from populations of Caucasian, Asian, Hispanic, and African ancestry. We present case reports of individuals with E50K and 691_692insAG mutations and discuss findings from more than a dozen studies that have carried out *OPTN* mutation screening.

## Methods

### Subjects

Informed consent was obtained from each participant according to a HIPAA-compliant study protocol approved by The University of Michigan Institutional Review Board for review of human subjects studies. Ophthalmologic examinations included slit-lamp biomicroscopy, optic disk examination, IOP by applanation, gonioscopy, and refraction. Individuals with known surgical or pharmacologic risk factors for glaucoma, such as steroid use, were excluded from this study.

OAG was diagnosed based on the presence of open filtration angles, glaucomatous optic discs and glaucomatous visual field changes. Individuals with elevated IOP, greater than or equal to 22 mmHg, were considered to have POAG if they showed adult-onset at 35 years of age or older, and to have JOAG if they showed onset prior to 35 years of age. They were deemed to have NTG if their highest known IOP never exceeded 21 mmHg.

Table 1 lists our study case and control subjects by their diagnosis and ancestry. Our 314 OAG subjects included 51 JOAG, 230 POAG, and 33 NTG cases. Our control samples came from 371 unrelated individuals. Normal control samples were matched for race to the cases, so that a sequence variant found in a particular case population had control screening carried out only in the control population of the same ancestry. In most cases, the sample screened was the proband of the family, but sometimes a different case from that family had to be used, such as when the proband was someone with an ambiguous diagnosis. For some families in which the proband had a sequence variant of interest, additional relatives were also screened. The group of normal control samples included samples from 48 individuals of African ancestry (Corielle Institute, Camden, NJ), 19 individuals of Hispanic ancestry, and 99 individuals of Asian ancestry (Corielle Institute) who had not been characterized for ophthalmologic phenotype. These uncharacterized population controls were used in a subset of experiments as detailed in Results.

**Table 1:** Ancestry and diagnosis of subjects.

**Population**	**JOAG**	**POAG**	**NTG**	**Total OAG**	**Control**	**Total**
African (U.S., Ghana, Nigeria, and the Caribbean)	14	63	4	81	88	169
Asian (Korea, China, and the Philippines)	2	1	2	5	117	122
Caucasian (Europe and the Middle East)	32	159	26	217	116	333
Hispanic (Mexico, Puerto Rico, Chile, Panama, and Colombia)	3	7	1	11	50	61
Totals	51	230	33	314	371	685

The frequency distribution of cases and controls according to open-angle glaucoma (OAG) condition and ancestry (national) in our analysis included 314 cases with juvenile onset OAG (JOAG), primary open-angle glaucoma (POAG), and normal tension glaucoma (NTG).

### Mutation Screening

*OPTN* was screened via sequencing of polymerase chain reaction (PCR) amplified DNA. Genomic DNA was extracted from peripheral blood samples using Puregene DNA Isolation kits (Gentra Systems, Minneapolis, MN) following the manufacurer's protocol. *OPTN* coding exons were amplified by PCR in a 20 μl reaction containing 50 ng of genomic DNA, 1.5 mmol/l MgCl_2_, 0.5 μmol/l of each primer, 0.125 mmol/l of each dNTP, and 0.5 units of Amplitaq Gold (PE Applied Biosystems, Foster City, CA) in 1X final concentration of the PCR buffer. Primers used for exon amplification are listed in Table 2. PCR conditions were 10 min at 95 °C followed by 36 cycles of 1 min at 95 °C, 1 min at 55 °C, and 1 min at 72 °C with a final extension for 10 min at 72 °C. Sequencing of PCR-amplified DNA was used to screen all 314 OAG cases for mutations in the coding sequences (i.e., exons 4 through 16) and splice sites flanking *OPTN* exons. When a sequence variant was detected in a patient, we screened for that specific mutation in the control population samples of the same ancestry.

**Table 2:** Primers used for sequencing the *OPTN* gene.

**Exon**	**Forward primer sequence (5'-3')**	**Reverse primer sequence (5'-3')**
4	TGGAGAGAAAGTGGGCAACT	CACCAGCTACCACCTATGGA
5	GGCATCTTTCAATTCAGAGCC	GACACGTAAGATTCCACTGC
6	TCCCAGAGCTCTGCGATTAA	GCTACACTGGAATTTCCTCA
7	TCTGAGCCACCCCGTTTAAA	GACCTCCGGTGACAAG
8	GGAGAATGTTCTGGAAAGCAG	GGGTGAACTGTATGGTATCT
9	CCCCTGATCCTTTATCCCAA	AATTCAGTGGCTGGACTAC
10	TGGTTCAGCCTGTTTTCTCC	CCCCCCATCTTACAAGTATTTC
11	TGGCCAGGTCTAGTGAAGAA	TTTATCCCCCTCTCTGAGAG
12	GAAATGCTAGTAGGTCGTGG	CCCTGACCATAGGACATTCA
13	CCGGCCAGAGCTGATAAT	AGATCCACTGAGCACTTTCC
14	CTAGCAGGATTGTGCATCGT	GTGGCGCGAACACAGCTATT
15	TTTCCCCTACTTCTGTGGAC	GAGACTGACGGGTGCTATAT
16	TCATGTCCCACTACGTGTTG	TGTGCCCGGCCTGTTTTCTT

Primers used in amplification of *OPTN* exons were also used in sequencing reactions. Primers located in introns were placed far enough away from the exon boundaries to allow visualization of the sequence of the splice sites. Exons 4 through 16 are the exons that contain coding sequence.

Individuals with E50K and 691_692insAG mutations who are presented in the case reports were screened for mutations in *MYOC*. PCR amplification of the three *MYOC* exons was conducted as described in reference [7] with some modified primers as listed in Table 3. PCR products were purified with a QIAquick PCR purification kit (Qiagen, Santa Clarita, CA). Sequenced PCR products were analyzed on an ABI 377 sequencer or at the University of Michigan DNA Sequencing Core facility on either an ABI 3730 or 3700 sequencer.

**Table 3:** Primers used for sequencing the *MYOC* gene.

**Exon**	**Forward primer sequence (5'-3')**	**Reverse primer sequence (5'-3')**
1A	GGCTGGCTCCCCAGTATATA	CTGCTGAACTCAGAGTCCCC
1B	AGGCCAATGTCAAGTCATCCAT	CTCCAGAACTGACTTGTCTC
2	ACATAGTCAATCCTTGGGCC	TAAAGACCACGTGGCACA
3A	CTGGCTCTGCCAAGCTTCCGCATGA	GGCTGGCTCTCCCTTCAGCCTGCT
3B	GAGCTGAATACCGAGACAGTGAA	GAGGCCTGCTTCATCCACAGCCAAC

Primers used in amplification of *MYOC* exons were also used in sequencing reactions. Primers located in introns were placed far enough away from the exon boundaries to allow visualization of the sequence of the splice sites. Primer pairs 1A, 2, and 3A were used for PCR amplification and sequencing of exons 1, 2, and 3, respectively. Primers 1B and 3B are internal primers that were used for sequencing purposes only.

### Statistical Analysis

Published reports on the frequency of OAG mutations were compared using several different statistical tests. Because some studies contained expected frequencies of less than 5, Fisher's exact test was chosen to examine the 2x2 contingency tables of individual studies. In tandem with Fisher's exact test, odds ratios and 95% confidence intervals for the odds ratio were calculated. To estimate odds ratios for whole populations based on multiple studies, fixed effect estimates were calculated using a Mantel-Haenszel (MH) model [25]. Homogeneity was evaluated with the Woolf test, in which the p value allows a determination of the appropriateness of combining studies by testing for evidence of effect modification by study group (i.e., testing whether the odds ratios are the same in all studies). For a case-control study, an odds ratio greater than 1 indicates that OAG cases are more likely to have the gene of interest than controls. For 2x2 contingency tables, independence is equivalent to an odds ratio of 1. All statistics were computed using the open source statistical program R 2.3.1 with the packages rmeta 2.12, meta 0.5, and vcd 0.9-7 [26-29].

## Results

### E50K *OPTN* mutation in a case with normal tension glaucoma

**Figure 1:**
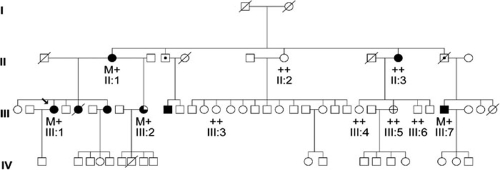
Lack of complete cosegregation of E50K with glaucoma in the pedigree of a Chilean family. The arrow indicates the proband (Case 1). Filled symbols are affected individuals with NTG, open symbols are individuals who are unaffected or reported to be unaffected. Symbols with a cross indicate individuals who are glaucoma-suspect, symbols with a center dot indicate glaucoma-affected individuals according to family report, and partially filled symbols denote individuals affected with POAG. Diagonal lines mark deceased individuals. Individuals denoted with ++ have E50 alleles on both chromosomes and ones with M+ carry the E50K heterozygous change. Members of generation four are young enough that they are not expected to be affected yet.

Case 1, a 52 year old Chilean female (III:1; Figure 1), was diagnosed with NTG at age 42 years and her highest pretreatment IOP was 18 mmHg in both eyes. After bilateral trabeculectomies and betaxolol treatment, her IOPs were 6 mmHg in the right eye and 10 mmHg in the left eye. Gonioscopic exam revealed open angles in both eyes, with iris processes noted circumferentially in both eyes. A dilated funduscopic exam demonstrated advanced glaucomatous cupping with cup-to-disc ratios of 1.0 in both eyes and absence of hemorrhage. Sequence changes were absent in the *MYOC* gene coding sequence and splice sites. Her family history showed evidence of autosomal dominant inheritance (Figure 1). The E50K mutation was found in three of the proband's four affected relatives that were in the study (II:1, III:2, and III:7; Figure 1). The E50K mutation was absent in the proband's affected aunt (II:3; Figure 1) as well as five unaffected relatives that were screened (Figure 1).

### 691_692insAG *OPTN* mutation in a case with primary open-angle glaucoma

Case 2 was a female Russian Ashkenazi Jewish immigrant diagnosed with POAG at 80 years of age. Her ocular history was significant for high myopia (right eye -17.75 diopters, left eye -19.00 diopters) and myopic retinal degeneration in both eyes. The patient had a childhood history of measles, a disease that has been identified as a possible contributor to high myopia [30,31]. At the time of the POAG diagnosis, the patient already had dense, 4-quadrant visual field defects in both eyes, attributable to the retinal degeneration, or a long-standing undiagnosed glaucoma, or both. She had cup-to-disc ratios of 0.5 in both eyes and diffuse chorioretinal atrophy. Her IOPs were 22 mmHg in the right eye and 21 mmHg in the left eye. Her IOPs decreased to 17 mmHg in the right eye and 16 mmHg in the left eye at one month after treatment with betaxolol, dipivefrin, and pilocarpine. Over the next several years, her IOPs fluctuated between the low teens and mid-twenties while she underwent treatment with medication, laser treatments, and multiple trabeculectomies. Screening of the *OPTN* gene revealed an insertion of AG at positions 691 and 692 (691_692insAG) in one copy of the *OPTN* gene. There were no other sequence variants in the coding sequence or splice sites of either *OPTN* or *MYOC.* Little family history information was available. She had a maternal grandfather affected by high myopia, but she didn't know of any other cases of glaucoma in her small family. Her only living relative, a reportedly unaffected son, declined to participate in the study.

### R545Q and M98K *OPTN* sequence variants

The R545Q sequence variant was found in two individuals with OAG. One woman with Filipino ancestry had JOAG diagnosed at 24 years of age and had a maximum known pretreatment IOP of 50 mmHg. Her mother also had POAG and her brother is unaffected. She did not know the diagnostic status of the rest of her relatives in the Philippines. The second case, a Korean woman diagnosed at 55 years of age, had a maximum known IOP of 29 mmHg and an unknown family history of glaucoma.

Among 36 OAG cases with the M98K mutation, the 26 for whom we have historical IOP data had known maximum IOPs between 16 mmHg and 55 mmHg (mean=29.6 mmHg). Out of the 283 OAG cases who lacked the M98K mutation, 253 had historical IOP data available, with the maximum recorded IOPs ranging from 14 mmHg up to 77 mmHg (mean=29.9 mmHg).

### *OPTN* sequence variants in the whole study cohort

Among the 314 OAG cases, we found a total of four (1.2%) individuals who possessed any of the three sequence variants reported by Rezaie and colleagues [9] to be disease-causing variants (Table 4).

**Table 4:** Frequency of sequence variants that alter the *OPTN* protein sequence.

**Protein change**	**DNA change**	**Exon**	**Ancestry**	**Cases**	**Controls**
E50K	c.458 G>A	4	Hispanic	1/11	0/50
I88V	c.572 A>G	5	Caucasian	0/217	1/116
A99S	c.605 G>T	5	African	0/81	2/88
E322K	c.1274 G>A	10	African	1/81	6/88
E322K	c.1274 G>A	10	Caucasian	1/217	0/90
691Frameshift	691_692insAG	6	Caucasian	1/217	0/116
R545Q	c.1944 G>A	16	Asian	2/5	11/117

All case samples were screened and scored for each mutation listed. Absence of a listing for one of the four control groups does not imply that it was screened. Data for M98K appear in Table 5.

The E50K mutation in Case 1 was the only instance of E50K among the 314 OAG cases (1/314, 0.3%) and in none of 371 controls (<2.7%), and was one of only 11 Hispanic cases screened (1/11, 9.1%; Table 4). E50K was identified in one of the 33 NTG cases (3.0%), but was not present in the 230 POAG or 51 JOAG cases. This mutation was present in 1/11 (9.1%) Hispanic cases and none of 50 Hispanic controls. It was also absent from 86 Caucasian normal controls.

The single instance of 691_692insAG in Case 2 was found among the 314 OAG cases (1/314, 0.3%), and was one of 217 Caucasian OAG cases screened (0.5%; Table 4). This was one of 230 POAG cases (0.4%) and was not present in 116 Caucasian controls, including seven samples that share Ashkenazi Jewish ancestry with the case having the mutation. In addition, 691_692insAG was present in one case in the original report by Rezeai [9] but that report did not provide information on ancestry of that case, so we could not tell whether their case and our case shared ancestral origins. This mutation was also previously reported as being absent from 200 normal control chromosomes of Caucasian origin [9].

R545Q was present in two of the 314 OAG cases (0.6%; Table 4). It was found once among the 51 JOAG cases (1.9%) and once among the 33 NTG cases (3.0%). Both instances of R545Q were found in individuals of Asian ancestry (2/5, 40.0%). R545Q was also present in 11 of 117 (9.4%) controls of Asian ancestry. The absence of this allele from our Caucasian cases or controls (0/333, <0.3%) concords with previous reports that failed to find it in either cases or controls of European ancestry (0/1457), suggesting that the allele frequency in European populations may be less than 0.1% [9,11,13,22,23,32,33]. Based on our data, we suggest that R545Q may be of low prevalence in African (<0.6%) or Hispanic populations (<1.6%), but our ability to estimate frequencies in these populations is limited because of sample size.

The M98K sequence variant was found in JOAG, POAG, NTG, and control populations (Table 5 and Table 6). We observed statistically insignificant differences in the frequencies between cases and controls for Africans and Caucasians, the two large sample sets in the study (Table 5). Frequencies in Asian samples (32/122, 26.2%) resembled values for African samples (18/81, 22.2%), while frequencies for Hispanic samples (2/62, 3.2%) seemed more similar to frequencies for Caucasian samples (8/116, 6.9%), but sample sizes were small. Additionally, the Asian samples showed no difference between cases and controls (p value=0.112), but the sample size was small and the use of a predominantly Chinese population to control for findings from a mixed Asian case set was problematic when looking at an allele that showed considerable variation among populations. Thus, although our overall study population showed M98K in 36 of 314 OAG individuals (11.5%) and 58 of 371 controls (15.6%), such pooling of data from different racial/ethnic groups is invalid where population frequencies vary so greatly.

**Table 5:** Frequency of M98K in four populations within our cohort.

**Ancestry**	**Whole population**	**JOAG**	**POAG**	**NTG**	**Total OAG**	**Controls**	**Fisher’s exact test p value**
Frequency of mutation in screened samples by population							
African	38/169	3/14	14/63	1/4	18/81	20/88	1.0
Asian	32/122	1/2	1/1	1/2	3/5	29/117	0.112
Hispanic	2/62	0/3	1/7	0/1	1/11	1/50	0.331
Caucasian	22/336	3/32	11/159	0/26	14/217	8/116	1.0
Total	94/690	7/51	27/230	2/33	36/314	58/371	
Percent of mutation in screened samples by population							
African	22.5	21.4	22.2	25.0	22.2	22.7	
Asian	26.2	50.0	100.0	50.0	60.0	24.8	
Hispanic	3.2	0.0	14.3	0.0	9.1	2.0	
Caucasian	6.5	9.4	6.9	0.0	6.5	6.9	
Total	3.6	3.7	1.7	6.1	1.5	5.6	

The total enumeration of both cases and controls is listed in the whole population column. Cases are subdivided according to OAG type, either JOAG, POAG, or NTG. A two-sided Fisher's exact test p value indicated no statistical significance for association between cases and controls in each ancestry category. Woolf's test for homogeneity among the ancestry frequencies yielded a p value of 0.312, indicating that the ancestral subdivisions are statistically similar.

**Table 6:** Comparison of R545Q frequency in different populations.

**Source**	**OAG**		**Controls**		**Percent**		**Odds ratio**	**95%CI bounds**		**Fisher's exact test p value**
	**R545Q**	**Total**	**R545Q**	**Total**	**OAG**	**Controls**		**Lower**	**Upper**	
China										
[14]	5	118	5	150	4.2	3.3	1.28	0.36	4.54	0.753
[15]	27	400	19	262	6.8	7.3	0.92	0.50	1.70	0.876
Japan										
[11]	12	247	3	89	4.9	3.4	1.46	0.40	5.31	0.767
[16]	26	411	11	218	6.3	5.0	1.27	0.62	2.62	0.596
[17]	1	154	0	100	0.6	0.0	1.96*	0.08	48.69*	1.000
[20]	20	313	10	196	6.4	5.1	1.27	0.58	2.77	0.700
[21]	3	83	4	58	3.6	6.9	0.51	0.11	2.35	0.446
Asia										
[This study]	2	6	11	117	33.3	9.4	4.82	0.79	29.36	0.122
Europe										
[This study]	0	217	-	-	0.0	-	-	-	-	-
[11]	0	650	0	162	0.0	0.0	-	-	-	-
[13]	0	27	0	94	0.0	0.0	-	-	-	-
[9]	1	46	0	100	2.2	0.0	6.63*	.26*	165.80*	0.315
[22]	0	112	-	-	0.0	-	-	-	-	-
Africa										
[This study]	0	81	0	90	0.0	0.0	-	-	-	-
India										
[19]	6	200	0	200	3.0	0.0	13.40*	0.75*	239.49*	0.030
Mixed										
[23]	0	86	0	80	0.0	0.0	-	-	-	-
[24]	1	114	3	187	0.9	1.6	0.54	0.06	5.28	1.000

The asterisks denote a calculation based on adding 0.5 to each cell in cases with a zero cell frequency, otherwise the value is nonexistent. Lower and upper bounds refer to the individual study 95% confidence interval around the odds ratio for a fixed effects Mantel-Haenszel model. Information from Leung et al. [35] were omitted because it duplicated that contained in Fan et al. [15]. Data from Toda et al. [37] were omitted because it duplicated information contained in Tang et al. [20]. In the Europe category, only the data regarding Caucasians (Iowa and Australia) in Alward et al. [11] were enumerated, while the reported cases with pigmentary, developmental, and exfoliative data were omitted.

Screening identified two instances of E322K in both cases and controls - a change previously reported to be associated with glaucoma [9] (Table 4). We also found silent *OPTN* coding sequence polymorphisms, including L32L, T34T, L41L, E63E, A134A, and S321S, as well as previously reported intronic sequence variants [4,10,11,20,24,34,35]. We did not find Ile88Val or Ala99Ser in our case populations, but did observe them among our controls (Table 4).

### Pooled data on R545Q

**Figure 2:**
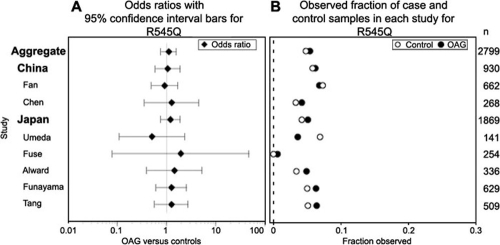
R545Q log odds ratios and allele frequences in Asian population studies. **A** shows the odds ratios with 95% confidence interval bars for individual Asian studies, and pooled results for Japan, China, and both in open angle glaucoma (OAG) cases versus controls. Odds ratios and confidence intervals are fixed effect estimates resulting from the Mantel-Haenszel method. **B** shows the case (OAG, filled circle) and control (open circle) proportion observed for each study. Total sample sizes are listed along the right-hand margin. None of the differences between case and control frequencies are statistically significant in a comparison of the odds ratios (as readily observed from the odds ratio confidence intervals) and frequencies of R545Q mutations in any of the Asian populations studied.

R545Q was present in our Asian data set, but absent from the other three populations we screened. We found no significant differences in allele frequencies between cases and controls for R545Q. This agrees with many of the other published studies, although it should be noted that Mukhopadhyay et al. [19] did report the case-control difference to be significant (Table 6). Evaluation of odds ratios and frequencies for each of the Asian studies showed no statistically significant differences between case and control values for any of the Asian data sets (Figure 2, Table 6). With the exception of the Rezaie study [9], R545Q has been reported only in Chinese, Japanese, Korean, Filipino, Indian, and mixed-ancestry populations (Table 6).

Our Asian data set is small, so we examined the possibility of pooling data from multiple studies. Because of observed variation in allele frequencies among studies (0.6-6.8%; Table 6), we questioned whether the data could be validly combined. Using the Woolf test for heterogeneity to address this question, we found no statistically significant study-based stratification within the individual populations for R545Q (Table 7). This means that it is reasonable to take data from the studies in Table 6 and pool them for a given population from multiple studies under a model of homogeneity. When pooled, we found no statistically significant difference between case and control values for the combined Asian data set (p value=0.541), nor for the separate Chinese (p value=0.89) or Japanese (p value=0.43) subsets (Table 7). The same lack of difference was true when considering only NTG cases.

**Table 7:** Aggregate statistical summaries in Asian populations screened for R545Q.

**Ancestry**	**Cases**		**Controls**		**Percent**		**Odds ratio**	**95% CI bounds**		**Woolf test p value**	**Fisher’s exact test p value**
	**R545Q**	**Total**	**R545Q**	**Total**	**OAG**	**Control**		**Lower**	**Upper**		
China	32	518	24	412	6.2	5.8	0.98	0.57	1.70	0.648	0.89
Japan	62	1208	28	661	5.1	4.2	1.20	0.76	1.90	0.838	0.43
Asia	94	1726	52	1073	5.4	4.8	1.12	0.78	1.58	0.925	0.541
China-NTG	7	106	5	150	6.6	3.3	2.04	0.54	8.41	-	0.244
Japan-NTG	40	705	28	661	5.7	4.2	1.40	0.84	2.33	0.848	0.263

Results from computing the upper and lower 95% confidence interval bounds around the odds ratio indicate that none of the Asian divisions are statistically different from an odds ratio of 1. The Woolf test for homogeneity indicates that across studies within each ancestry group the odds ratios are statistically equivalent (i.e., homogeneous, because a p value less than 0.05 would indicate heterogeneity [25]). A two-sided Fisher's exact test on the pooled frequencies was computed for those instances when the Woolf test indicated homogeneity at the 0.05 level. The ancestry groups are collated for China from Chen et al. [14] and Fan et al. [15]; and for Japan from Alward et al. [11], Funayama et al. [16], Fuse et al. [17], Tang et al. [20], and Umeda et al. [21]. The Asia listing includes data pooled from the China and Japan categories. Only Fan et al. [15] report on NTG for China.

### Pooled data on M98K

We found no evidence of significant difference between case and control frequencies for M98K in our Asian, African, Hispanic, or Caucasian populations (Table 8). Fuse and Alward reported statistically significant evidence of association of M98K with OAG in the Japanese population, although Alward indicated that this difference becomes nonsignificant when adjusted for testing multiple times [11,17]. Other studies do not report a significant case-control difference [11-24].

**Table 8:** Frequency of M98K in individuals from different populations.

**Source**	**Cases**		**Controls**		**Percent**		**Odds ratio**	**95% CI bounds**		**Fisher’s exact test p value**
	**M98K**	**Total**	**M98K**	**Total**	**OAG**	**Control**		**Lower**	**Upper**	
China										
[14]	26	118	22	150	22.0	14.7	1.64	0.88	3.08	0.148
[15]	129	400	81	281	32.3	28.8	1.18	0.84	1.64	0.355
Japan										
[11]	51	247	8	89	20.6	9.0	2.63	1.20	5.80	0.014
[16]	81	411	36	218	19.7	16.5	1.24	0.81	1.91	0.389
[17]	25	154	5	100	16.2	5.0	3.68	1.36	9.97	0.009
[20]	51	313	27	196	16.3	13.8	1.22	0.74	2.02	0.527
[21]	12	83	1	58	14.5	1.7	9.63	1.22	76.31	0.149
Asia										
[This study]	3	5	29	117	60.0	24.8	4.55	0.72	28.60	0.112
Europe										
[This study]	13	217	8	116	6.0	6.9	0.86	0.34	2.14	0.814
[11]	46	650	10	162	7.1	6.2	1.16	0.57	2.35	1.000
[12]	22	315	3	95	7.0	3.2	2.30	0.67	7.87	0.224
[13]	2	27	3	94	7.4	3.2	2.43	0.38	15.33	0.310
[32]	9	200	10	200	4.5	5.0	0.90	0.36	2.25	1.000
[18]	11	237	5	110	4.6	4.5	1.02	0.35	3.02	1.000
[33]	11	170	1	100	6.5	1.0	6.85	0.87	53.87	0.036
[9]	23	169	9	422	13.6	2.1	7.23	3.27	15.98	0.000
[22]	7	105	7	93	6.7	7.5	0.88	0.3	2.6	1.000
Hispanic										
[This study]	1	11	1	50	9.1	2.0	4.90	0.28	85.05	0.331
Africa										
[This study]	18	81	20	88	22.2	22.7	0.97	0.47	2.00	1.000
India										
[19]	22	200	11	200	11.0	5.5	2.12	1.00	4.51	0.068
[39]	10	220	0	100	4.5	0	inf	1.04	inf	0.034
Mixed										
[38]	28	498	17	218	5.6	7.8	0.70	0.38	1.32	0.315
[36]	14	153	9	100	9.2	9.0	1.02	0.42	2.45	1.000
[23]	8	86	8	80	9.3	10.0	0.92	0.33	2.59	1.000
[24]	12	115	4	101	10.4	4.0	2.83	0.88	9.06	0.116

Under the fixed effects Mantel-Haenszel model, individual study 95% confidence interval bounds around the odds ratio are given. For compatibility with much of the published literature the two-sided Fisher's exact test p values are given for each study. Although the presence of M98K mutations in OAG cases appears to be statistically significant relative to controls, Alward et al. [11] reported that when multi-testing is taken into account their result becomes nonsignificant. Information from Leung et al. [35] was omitted because it duplicated the information contained in Fan et al. [15]. Data from Toda et al. [37] were omitted because they duplicated the observations contained in Tang et al. [20]. Pigmentary and exfoliative data were omitted from the Europe (Caucasians living in Iowa and Australia) samples reported by Alward et al. [11]. Rezaie et al. [9] reported a p value=2.18^-7^.

Previous studies supported our finding that M98K allele frequences are much higher in populations of Asian (555/2818, 19.7%) or African (38/169, 22.5%) ancestries than in Caucasian (179/3149, 5.7%) or Hispanic (2/61, 3.3%) populations (Table 8 and Table 9). Thus, ancestry would be a significant confounding variable when attempting to analyze data pooled from different populations.

**Table 9:** Aggregate statistical summaries for populations screened for M98K.

**Ancestry**	**OAG**		**Controls**		**Percent**		**Odds ratio**	**95% CI bounds**		**Woolf test p value**	**Fisher's exact test p value**
	**M98K**	**Total**	**M98K**	**Total**	**OAG**	**Controls**		**Lower**	**Upper**		
China	155	518	103	431	29.9	23.9	1.26	0.94	1.70	0.354	0.04
Japan	220	1208	77	661	18.2	11.6	1.65	1.25	2.19	0.046	-
Asia	375	1726	180	1092	21.7	16.5	1.46	1.19	1.79	0.075	0.0006
Europe	131	1873	48	1276	7	3.8	1.87	1.31	2.66	0.005	-

Total	600	4871	277	3167	12.3	8.7	1.51	1.29	1.77	0.002	-

Japan-NTG	142	705	77	661	20.1	11.6	1.91	1.40	2.62	0.240	2E-05
Europe-NTG	28	371	24	544	7.5	4.4	1.77	0.97	3.24	0.149	0.58
Total-NTG	170	1076	101	1205	15.8	8.4	1.75	1.33	2.31	0.177	6E-08

The ancestry groups are collated for China: Chen et al. [14] and Fan et al. [15]; Japan: Alward et al. [11], Funayama et al. [16], Fuse et al. [17], Tang et al. [20], and Umeda et al. [21]; Europe: Alward et al. [11], Aung et al. [12], Baird et al. [13], Jansson et al. [32], Melki et al. [18], Rakhmanov et al. [33], Rezaie et al. [9], and Weisschuh et al. [22]. Asia is the pooling of the China and Japan categories. The total combines all published studies from the lines above with the addition of Craig et al. [38], Hauser et al. [36], Mukhopadhyay et al. [19], Sripriya et al. [39], Wiggs et al. [23], and Willoughby et al. [24]. The Europe-NTG group consists of the data from Alward et al. [11], Aung et al. [12], Baird et al. [13], Rakhmanov et al. [33], and Weisschuh et al. [22]. Results from computing the upper and lower 95% confidence interval bounds around the odds ratio indicate that some studies are statistically different than an odds ratio of 1. The Woolf test for homogeneity indicated that across studies within each ancestry group which of the odds ratios were statistically equivalent (i.e., heterogeneity is indicated by a p value less than 0.05 [25]). A two-sided Fisher's exact test on the pooled frequencies is given for those instances when the Woolf test indicated homogeneity at the 0.05 level. When Rezaie et al. [9] data are excluded the Europe group, then the odds ratio becomes 1.33 with a 95% confidence interval of (0.90, 1.98), Woolf p value of 0.512, and a Fisher's exact test p value of 0.072.

**Figure 3:**
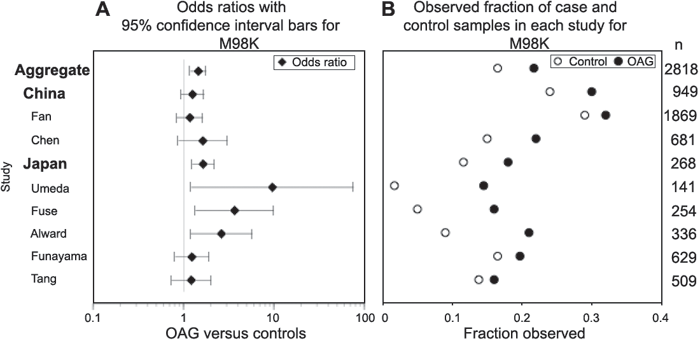
Studywise differences appear in Japanese populations when odds ratios and frequencies of M98K mutations are compared. The left-hand graph (**A**) shows the odds ratios with 95% confidence interval bars for individual Asian studies and pooled results for Japan, China, and both in open angle glaucoma (OAG) cases versus controls. Odds ratios and confidence intervals are fixed effect estimates resulting from the Mantel-Haenszel method. The right-hand graph (**B**) shows the case (OAG, filled circle) and control (open circle) proportions observed for each study. Total sample sizes are listed along the right-hand margin. Larger samples have both narrower confidence intervals and shorter distance between fractions observed for cases and controls. Studies inconsistently estimate the odds of OAG versus controls carrying an M98K mutation, with larger studies (more than 400 total cases and controls) estimating no statistically significant difference. Other population estimates are not shown, because, among the European population-based studies, only Rezaie's study [9] showed a statistically significant difference. The single study on India yielded a significant odds ratio, but no other comparable populations have been reported [19].

If we consider specific defined subpopulations, where there should be less concern about ancestry serving as a confounding variable, then we are left with concerns about differences observed not just between but also within populations. When we compared the different Asian studies using odds ratios (Figure 3), the aggregate Asian data, the aggregate Japanese data, the data produced by our study, and by the individual studies of Umeda et al. [21], Fuse et al. [17], and Alward et al. [11], we found each showed significant evidence of a difference between cases and controls. When we evaluated allele frequencies for the various Asian data sets, we saw dramatic differences in allele frequencies among the different studies (Figure 3, Table 8). Also, the control values showed much greater variation among studies than the case values. The control values from some studies are higher than the case values from other studies, even though within each separate study the case frequencies are always higher than control frequencies (Figure 3). With regard to our study, in which a small number of samples came from diverse Asian regions, the observed difference could be due to the limited case sample size, differential representation of M98K within the Asian population, case versus control status, or some combination of the three.

The results of the Woolf test for heterogeneity indicated that there was something noncomparable about the M98K findings from a number of the studies that reported results for the same populations (Table 9). In the case of the Japanese data set, pooled data showed M98K frequencies of 18.2% (220/1208) in cases versus 11.6% (77/661) in controls (p value=0.0002), but results of the Woolf test led us to suspect that we may be combining noncomparable data sets (p value=0.046). In the case of the European data set, Fisher's exact test indicated that there was a statistically significant difference between cases and controls (p value=0.001), but again the Woolf test identified heterogeneity among the data sets (p value=0.005; Table 9). When we pooled the worldwide data from the entire set of published studies, we saw a significant difference between cases and controls (p value=0.00000004), but again, testing for heterogeneity indicated that it may be invalid to pool these studies (Table 9; p value=0.0018). Interestingly, if we removed data from the Rezaie study [9], which indicated Caucasian controls but did not specify its case population composition, the remaining Caucasian data sets appeared to be homogeneous (p value=0.512) and Fisher's exact test indicated no significant difference between case and control values (Table 9; p value=0.072). Fewer studies distinguish observations for NTG only. For those studies that provided data for NTG frequencies, we saw that both the Japanese and European populations showed homogeneity across studies. However, Fisher's exact testing of pooled studies showed opposing results between Japanese (p value=0.00002) and European (p value=0.58) populations.

The case versus control difference for the Hispanic data are also intriguing, with M98K case values of 1/11 (9.1%) and control values of 1/50 (2.0%). Our small Hispanic data set was not well-powered for statistical testing, but this represented an initial view of a population under represented in previous studies (Table 8). Given that our Hispanic data set represented cross-continental cases from North, Central, and South America, we have to wonder whether the apparent differences in M98K allele frequences was simply due to small sample size, or rather might be attributed to differential allele frequencies correlated with geographic origins of samples rather than case-control status.

## Discussion

In our study of 314 individuals with OAG, we found 42 individuals with sequence variants predicted to alter the protein coding sequence. This included three *OPTN* variants previously reported to be disease-causing variants-E50K, 691_692insAG, and R545Q, as well as the M98K variant previously reported as a risk factor [9].

This is the second time the 691_692insAG mutation has been reported. Both times it has been identified in case populations but not seen in controls. It is the first report of 691_692insAG in an individual of Russian Ashkenazi Jewish ancestry, and ancestry is unavailable for the previously reported case. Although *OPTN* defects were originally reported in a population of primarily NTG cases, we found this variant in an individual with modestly elevated IOP (22 mmHg). The shift in the reading frame that it causes and the fact that it is seen among cases but not controls suggests that it could be a causative variant. However, if we combine data from all studies, we see it in 2/3677 cases (0.0005%) in the studies that used protocols that would have detected it (all studies in Table 8 except Aung [12], Melki [18], and Wiggs [23]). Only a fraction of the 2,270 controls from those studies screened the whole sequence from all controls, so they would not have been highly likely to detect this variant among the controls even if the control frequency were equal to the case frequency. Thus, while it is tempting to say that a variant seen only in two cases might be causative, the available numbers can only support the conclusion that 691_692insAG event is a rare occurence.

We report here the first observation of the E50K change in a Caucasian Hispanic individual. The observation of E50K at a frequency of 0.3% in our cases is consistent with reports of frequencies in OAG populations of 0.1% by Alward et al. [11], 0.6% by Aung et al. [12], and 0.6% by Hauser et al. [36]. The NTG subset is reported to have E50K at a higher prevalence of 13.5% (7/52),1.5% (2/132), 1.5% (1/67), and 2.9% (1/34) in studies by Rezaie et al. [9], Aung et al. [12], Hauser et al. [36], and ourselves, respectively. Many other studies found no evidence of this mutation, including reports of its absence from 237 cases with Chinese ancestry [14,35] and 961 cases with Japanese ancestry [11,14,16,17,20,21,35,37], which supports the supposition that this is a polymorphism private to the Caucasian and Hispanic populations. Variation in frequencies observed among studies may be affected by the ancestry of the population, the fraction of the cohort with familial glaucoma, and differences in specific diagnoses included in the study. Failure to see complete cosegregation of E50K with glaucoma (i.e., we have one OAG case in a family lacking the E50K mutation) raises questions about whether we are observing a phenocopy or whether E50K is not the cause of the glaucoma in this family.

Our data and the compiled evidence from more than a dozen other studies support the idea that R545Q may be a private polymorphism of Asian populations. Although our Asian data set provides marginal evidence for a difference in R545Q allele frequency between cases and controls, it is a small population and the results are not statistically significant. When we pooled our data with data from other studies there did not appear to be any evidence to support a role for R545Q as a disease causing variant.

We found the M98K variant in all four populations screened, but evaluation of all of the published studies leaves unresolved the issue of whether or not M98K is a risk factor for glaucoma. A similar conclusion was drawn by Craig et al. [38] but they did not analyze the population (ancestry) structure of the data for the allele. Several studies find evidence for association, while others do not. Evaluation of the published data in addition to our own indicates that there is considerable variation in allele frequencies, not only among populations, but also within populations. This variant is found in Asian and African populations at more than twice the frequency seen in Caucasian and Hispanic populations. Comparison of findings from different studies indicates large variations in allele frequencies in different study populations within Japanese (MH p value=0.00038) and Chinese (MH p value=0.118) populations (Table 9). The case-control difference is much smaller within Caucasian or African populations (Figure 3), and data on Caucasian populations show less variation between studies than the data on Japanese and Chinese populations (Table 9, Figure 3).

There are a number of confounding factors that might contribute to the observed variability of allele frequency between Asian populations. Differential allele frequencies within a population could result from founder effects. At this point, there is not enough information available regarding origins of the different subpopulations (Table 10) to allow for evaluation of the likelihood of a founder effect. There appears to be a correlation between total sample size and the difference between case and control frequencies (Figure 2), although the published studies seem to be adequately powered (for Fisher's exact test, the power, or probability to reject the null hypothesis when it is true, is 0.76 for the parameters π_1_=0.2, π_2_=0.1, where each sample size is 200 and α=0.05). An alternative confounding factor could be the result of the different screening techniques applied (Table 10); however, there is no obvious correlation of high allele frequencies with one screening approach and low allele frequencies with a different technique. Selective under-representation of an allele in a data set relative to the actual allele frequency could result if M98K were in linkage disequilibrium with a neighboring polymorphism contained within the sequence of a primer used in amplification or sequencing in some studies, but not others. Some of the papers do not present the primer sequences and the available primer sequence data do not provide support for this idea. Additional contributions to variability between studies could include differences in diagnostic inclusion and exclusion criteria and fraction of familial glaucoma within each cohort. Thus, the extant data do not allow us to distinguish between technical, ascertainment and demographic models for the observed differences in M98K allele frequencies between different studies of the same population.

**Table 10:** M98K population data sources and screening methods ordered by ancestry.

**Source**	**Recruitment locations (population)**	**Methodology**	**Notes**
China			
[14]	China-Beijing	SSCP->sequencing	
[15]	China-Hong Kong	PCR and HTCSGE->sequencing	
Japan			
[11]	Japan-Gifu	SSCP->sequencing	
[16]	Japan-Tokyo, Kumamoto, Hamamatsu, Hiroshima, Niigata	PCR-RFLP	
[17]	Japan-Miyagi	PCR->sequencing	
[20]	Japan-Yamanashi	SSCP->sequencing	
[21]	Japan-Okayama City	sequencing	
Asia (other than China and Japan)			
[This study]	USA-Michigan (Korean, Chinese, Filipino)	PCR->sequencing	
Europe			
[This study]	USA-Michigan	PCR->sequencing	Caucasian
[11]	Australia-Melbourne, Adelaide, USA-Iowa	SSCP->sequencing	Australian samples Are Caucasian (D. Mackey, personal report), Iowa population >91% Caucasian according to the State Data Center of Iowa
[12]	England-London	PCR-RFLP	Caucasian
[13]	Australia-New South Wales	PCR-RFLP	mostly Caucasian
[32]	Sweden-Uppsala and Tierps	DHPLC, PCR, SNaPshot	
[18]	France-Paris	PCR-RFLP	French and Moroccan Caucasians
[33]	Russia-St. Petersburg	SSCP, PCR	
[9]	USA-Chicago, Connecticut, New Haven, UK-London, Canada-Toronto	PCR->sequencing and SSCP->sequencing	Unspecified cases with Caucasian controls
[22]	Germany-Tuebingen, Wuerzburg	PCR-RFLP, DHPLC	
Hispanic			
[This study]	USA-Michigan, Florida, Mexico, Panama, Peru, Chile, Paraguay	PCR->sequencing	
Africa			
[This study]	USA-Michigan, Ghana- Acra, Sunyani	PCR->sequencing	African American and African
India			
[19]	India-Hyperabad, Kolkata	SSCP->sequencing, DHPLC, PCR--RFLP	
[39]	India-Chennai	PCR->sequencing, RFLP	
Mixed			
[38]	Australia/Tasmania	PCR-RFLP	
[36]	USA-New England area	PCR->sequencing, DHPLC	about 90% Caucasian
[23]	USA-Massachusetts, North Carolina	PCR->sequencing	about 90% Caucasian
[24]	Canada-Toronto	PCR-RFLP	

The following methodologies were used to screen samples for variants. We omitted two studies: Wang et al. [40], a Filipino population, and Forsman et al. [34], a population from south Finland, because they are small family-based studies without population data. SSCP: single-strand conformation polymorphism. DHPLC: denaturing high-performance liquid chromatography. HTCSGE: High throughput conformation sensitive gel electrophoresis. RFLP: Restriction Fragment Length Polymorphisms.

An alternative approach to evaluation of whether the M98K allele is involved in glaucoma is through the study of cosegregation in families. Wiggs et al. [23] reported lack of cosegregation in families. An accompanying population-based portion of that study [23] also failed to find association of M98K with glaucoma in a population of mostly European ancestry.

One alternative explanation for why some studies find association, yet others do not, might be that there is a valid statistical difference between the case and control populations but that the M98K allele is actually associated with some other variable that differs between cases and controls, or has been excluded from cases but not controls. An obvious example of this would be IOP. The Rezaie et al. study [9] looked at mostly NTG families, while there was a lot of variation in the extent to which NTG was represented in the different populations in the other studies. If M98K is actually responsible for reducing IOP, or for preventing the rise of IOP, but is not actually causing glaucoma, then we would expect exclusion of individuals with elevated IOP from the cases would bias the M98K frequency in the cases as compared to the controls even if M98K were not actually causing glaucoma. Melki et al. [18] offered the view that M98K is associated with lower IOP. In our data set we found that cases with the M98K mutation had known maximum IOP values ranging from 16 mmHg to 55 mmHg (mean=29.6 mmHg) while those who lacked the M98K mutation had maximum recorded IOP values ranging from 14 mmHg in an NTG case up to 77 mmHg (mean=29.9 mmHg). Thus, in our data set there was no obvious difference in maximum known IOP between those with and those without M98K.

The original report by Rezaie provided apparently compelling statistical evidence that M98K is a glaucoma risk factor with a p value of 2.18x10^-7^. The other studies that found any support for M98K association with glaucoma found only weak evidence of this association. It remains unclear what the differences are between the studies that could account for the differences in study outcome. One key issue for M98K appears to be the great variability of allele frequency reported in different studies and different populations. This would be a problem if the case population in the Rezaie et al. study [9] contained multiple populations or an admixed population that self-identified as Caucasian. In the Rezaie et al. study [9], both E50K, an apparently private Caucasian polymorphism, and R545Q, an apparently private Asian polymorphism, were in the same study cohort. While this could mean that their undescribed case population was a mixed race group, it is also the kind of thing that can happen in a fairly admixed urban population when using self identification as the basis for applying racial/ethnic classification, even when setting out to identify a relatively homogeneous population. Thus, there is the possibility that the Rezaie et al. study [9] outcome differs from the others because of differences in diagnostic inclusion and exclusion criteria or other unidentified factors, but it could also be the result of studying an allele that varies significantly between and within populations - something that can happen even when making efforts to carry out adequate matching of cases and controls.

Thus the findings on M98K are currently contradictory, with some studies finding association and other studies finding no support for association, and with the differences in study outcome not assorting according to population or technology used. Because there are such substantial differences in allele frequencies between the different studies and between and within populations, it is likely that a final resolution of this question will require the following: The screening to take place by technologies selected for precision rather than high throughput; the study be adequately powered; matching of cases and controls for ancestry be highly rigorous and matched for subpopulations rather than simply matching for one of a handful of racial or ethnic categories; inclusion and exclusion criteria be carefully defined, that tests for association with associated variables such as IOP be carried out in addition to tests for association with the primary glaucoma status variable; and population substructure analysis be included in the analysis to help deal with apparent differences within populations that can be difficult to control.

This study has contibuted additional evidence of association of *OPTN* E50K with glaucoma, and reported an additional instance of the 691_692insAG sequence variant. We have also provided new information on *OPTN* in populations of African and Hispanic ancestry. Evaluation of data from more than a dozen studies indicated no association of R545Q with glaucoma in most populations. Combined analysis of more than a dozen studies suggests that M98K is associated with NTG in Asian, but not Caucasian study populations, but these results must be interpreted with great caution because of the large differences in allele frequencies between and within populations.
